# The mutational repertoire of uterine sarcomas and carcinosarcomas in a Brazilian cohort: A preliminary study

**DOI:** 10.6061/clinics/2021/e2324

**Published:** 2021-01-11

**Authors:** Leonardo Tomiatti da Costa, Laura Gonzalez dos Anjos, Luciane Tsukamoto Kagohara, Giovana Tardin Torrezan, Claudia A. Andrade De Paula, Edmund Chada Baracat, Dirce Maria Carraro, Katia Candido Carvalho

**Affiliations:** I Laboratorio de Ginecologia Estrutural e Molecular, Disciplina de Ginecologia, Hospital das Clinicas (HCFMUSP), Faculdade de Medicina, Universidade de Sao Paulo, Sao Paulo, SP, BR; II School of Medicine, Sidney Kimmel Comprehensive Cancer Center, Johns Hopkins University, Baltimore, MD, USA; III Grupo de Biologia Molecular e Genomica, Centro A.C.Camargo, Sao Paulo, SP, BR

**Keywords:** Sarcoma, Carcinosarcoma, Mutation, DNA Sequence Analysis

## Abstract

**OBJECTIVES:**

The present study aimed to contribute to the catalog of genetic mutations involved in the carcinogenic processes of uterine sarcomas (USs) and carcinosarcomas (UCSs), which may assist in the accurate diagnosis of, and selection of treatment regimens for, these conditions.

**METHODS:**

We performed gene-targeted next-generation sequencing (NGS) of 409 cancer-related genes in 15 US (7 uterine leiomyosarcoma [ULMS], 7 endometrial stromal sarcoma [ESS], 1 adenosarcoma [ADS]), 5 UCS, and 3 uterine leiomyoma (ULM) samples. Quality, frequency, and functional filters were applied to select putative somatic variants.

**RESULTS:**

Among the 23 samples evaluated in this study, 42 loss-of-function (LOF) mutations and 111 missense mutations were detected, with a total of 153 mutations. Among them, 66 mutations were observed in the Catalogue of Somatic Mutations in Cancer (COSMIC) database. *TP53* (48%), *ATM* (22%), and *PIK3CA* (17%) were the most frequently mutated genes. With respect to specific tumor subtypes, ESS showed mutations in the *PDE4DIP*, *IGTA10*, and *DST* genes, UCS exhibited mutations in *ERBB4*, and ULMS showed exclusive alterations in *NOTCH2* and *HER2*. Mutations in the *KMT2A* gene were observed exclusively in ULM and ULMS. *In silico* pathway analyses demonstrated that many genes mutated in ULMS and ESS have functions associated with the cellular response to hypoxia and cellular response to peptide hormone stimulus. In UCS and ADS, genes with most alterations have functions associated with phosphatidylinositol kinase activity and glycerophospholipid metabolic process.

**CONCLUSION:**

This preliminary study observed pathogenic mutations in US and UCS samples. Further studies with a larger cohort and functional analyses will foster the development of a precision medicine-based approach for the treatment of US and UCS.

## INTRODUCTION

Sarcomas are rare heterogeneous tumors that affect the female genital tract and originate from tissues such as muscle, fat, bones, and fibrous tissue. Uterine sarcomas (USs) are the most commonly occurring gynecological sarcomas, representing 90% of the total cases (1). Based on their histological composition, uterine tumors with mesenchymal elements can be divided into 1) pure sarcomas (uterine leiomyosarcomas - ULMSs, endometrial stromal sarcomas - ESSs); 2) mixed epithelial and mesenchymal tumors (adenosarcomas - ADSs), and 3) carcinosarcomas - UCSs, a biphasic tumor composed of high-grade carcinomatous and sarcomatous components derived from transdifferentiation of carcinoma (2). Many studies have characterized UCS tumors as mixed USs; however, since 2014, they have been reclassified as endometrial carcinomas (ECs) that demonstrate metaplastic features (3,4). Despite their low prevalence, USs are associated with high rates of local recurrence, distant metastases, and poor prognosis, with two-year survival rates below 50% (1).

Several genetic alterations have been associated with USs and UCSs, with few alterations being associated with specific histological subtypes. For instance, ESSs can be divided into two types: low-grade ESS (LG-ESS) and high-grade ESS (HG-ESS), both characterized by recurrent chromosomal translocations. In LG-ESS, the most common translocation, t [7; 17] (p15; q21), is observed in almost 50% of the cases and results in the *JAZF1-SUZ12* gene fusion (5). Ma et al. (6), revealed that the JAZF1-SUZ12 fusion protein destabilizes polycomb repressive complex 2 (PRC2), abolishes histone methyltransferase (HMT) activity, and subsequently activates genes normally repressed by PRC2. JAZF1-PHF1, EPC1-PHF1, PHF1-MEAF6, MBTD1-CXorf67, and JAZF1-BCORL1 are other less frequent fusion proteins observed in the patients with these tumors. HG-ESS exhibits a *YWHAE-NUTM2* gene rearrangement (previously termed *YWHAE-FAM22*). Recently, molecular alterations in ZC3H7B-BCOR, BCOR-ITD, EPC1-BCOR, JAZF1-BCORL1, and BRD8-PHF1 have been identified. This histological subtype demonstrates more aggressive clinical behavior and worse prognosis (5,2). Many previous studies have investigated the ESS genome with a focus on genetic fusions (7-10). However, Choi et al. (11) demonstrated that fusions are not the only genetic alterations that occur during the development of ESS. Using whole-exome sequencing methods, the aforementioned study described mutations in *PTEN*, *RB1*, *TP53*, and *CDH1*. Despite the use of a very small number of ESS samples in this study (3 LG-ESS), it is a valuable contribution to the understanding of the pathogenesis of such tumors.

ULMSs are not characterized by specific chromosome translocations; however, they are associated with a complex karyotype with chromosomal gains and losses, such as deletion in chromosome 1. Most ULMSs express PDGFR-α, WT1, CYP19, and GNRH-R (12,13). Owing to gene alterations, the loss of function in the tumor suppressor genes, *BRCA1* and *MED12* as well as the loss of expression of the proteasome β1i subunit LMP2 have been associated with ULMS development (14). Additionally, The Cancer Genome Atlas (TCGA) Research Network (15) examined the molecular characterization of adult soft tissue sarcomas (STSs) and observed that ULMSs shared more similarities with extrauterine LMSs than that with other sarcomas. Although both tumors exhibit the same pattern of cell differentiation, their tumor environments are extremely diverse. This study included 53 cases of soft-tissue LMS (extrauterine) and 27 ULMS cases that were evaluated by whole-exome sequencing, demonstrating frequent alterations in *TP53*, *RB1*, *ATRX*, and *MED12* (16).

Somatic mutations have also been described occurring at low frequency in the majority of the tyrosine kinase growth factor gene family and their targets, namely, v-raf murine sarcoma viral oncogene homolog B1 (*BRAF*), *CDKN2A*, epidermal growth factor receptor (*EGFR*), *HER2,* v-kit Hardy-Zuckerman 4 feline sarcoma viral oncogene homolog (*KIT*), v-Ki-ras2 Kirsten rat sarcoma viral oncogene homolog (*KRAS*), platelet-derived growth factor receptor (*PDGFR*), and phosphatidylinositol-4,5-bisphosphate 3-kinase, catalytic subunit α (*PI3KCA*) during the development of UCS. In addition, mutations in *TP53*, *PTEN*, protein phosphatase 2 scaffold subunit alpha (*PPP2R1A)*, F-box, and WD repeat domain containing 7 (*FBXW7*) have already been identified, which may contribute to the development of therapeutic alternatives including the use of the inhibitors of PARP, EZH2, cell-cycle, and PI3K pathway (14,17). Little information is available on how mutations contribute to ADS etiology; however, one study observed that *DICER1* mutations are associated with the tumorigenic process in a small subset of such tumors (18).

Since these are rare tumors, only a few studies focusing on the definition of the mutational repertoire of the different histological types of rare sarcomas have been conducted thus far. Therefore, studies focusing on the mutational characterization of these tumors are of paramount importance and will contribute to the discovery of new biomarkers for precision medicine-based approaches in the treatment of such neoplasms. Herein, we investigated the mutational profile of the samples obtained from patients with ULMSs, UCSs, ESSs, and ADSs, using a commercial panel containing 409 cancer-associated genes involved in apoptosis, signaling, transcription regulation, inflammation response, and growth factors-associated pathway.

## MATERIALS AND METHODS

### Sample selection

In order to analyze differences in genetic mutations between different histological types of US, we initially selected 43 formalin-fixed and paraffin-embedded (FFPE) human samples including 14 ULMS, 12 ESS, 2 ADS, 12 UCS, and 3 ULM-non-cancerous tumor (as reference samples). All samples were obtained via surgical procedures performed between 2000 and 2012 at the Institute of Cancer of Sao Paulo (ICESP) and Clinics Hospital of the Faculty of Medicine, University of Sao Paulo (HCFMUSP). Tissues were stored at the molecular and structural gynecology laboratory (LIM-58) of the University of Sao Paulo Medical School (FMUSP).

This study was performed in accordance with the Declaration of Helsinki and was approved by the Research Ethics Committee of the FMUSP with protocol number 477/15. Patients' medical records were revised and the following data were recorded: age at diagnosis, postmenopausal bleeding, adjuvant treatment, presence of metastasis or recurrence, and status.

### DNA Isolation

Genomic DNA was extracted using the QIAamp DNA FFPE Tissue Kit obtained from QIAGEN^®^ according to the manufacturer’s instructions. DNA concentration, purity, and integrity were assessed by spectrophotometry (Nanodrop 2000, Thermo Fisher Scientific) and fluorometry (Qubit - Thermo Fisher Scientific), respectively.

### Preparation of sequencing libraries and Next-Generation Sequencing (NGS)

Sequencing libraries were prepared using the Ion Torrent Ampliseq Comprehensive Cancer Panel - Catalog number: 4477685 (Thermo Fisher Scientific), which contains ∼16,000 primer pairs multiplexed into 4 pools. This commercial panel was designed to assess the mutational profile of 409 cancer driver genes and drug targets along with signaling cascades, apoptosis genes, DNA repair genes, transcription regulators, inflammatory response genes, and growth factor genes (Table S1). Prior to amplification, DNA was treated with the uracil-DNA glycosylase enzyme (Thermo Fisher Scientific) by adding 1 unit of enzyme per 50 ng of DNA and incubating for 15 min at 37 °C. This procedure was performed to remove DNA molecules containing uracil and decrease the number of artifactual variants in the sequencing (19). Libraries were then prepared using Ion AmpliSeq^TM^ Library kit 2.0 protocols, with 10 ng of input DNA per pool, totaling 40 ng of DNA from each sample. The FuPa reagent was used to partially digest primer sequences and phosphorylate the amplicons. Next, sequencing adaptors and barcodes were ligated to the amplicon by the enzyme Ligase using the Ion Xpress™ Barcode Adapters kit (Thermo Fisher Scientific), which were then purified using magnetic beads (Agencourt^®^ AMPure^®^ XP Reagents, Beckman Coulter). Subsequently, emulsion PCR was performed using the Ion PI™ Hi-Q™ OT2 200 Kit (Thermo Fisher Scientific), followed by sequencing with Ion PI^TM^ Hi-Q^TM^ sequencing 200 and Ion PI^TM^ Chip.

### Data Analysis

The results were analyzed using the Torrent Suite v5.0.5 software (Thermo Fisher Scientific). Sequence variants (SNVs and indels) were identified using the Torrent Variant Caller (Ion Torrent - Thermo Fisher Scientific) and compared to the GRCh37 / hg19 genome version. VCF files were analyzed using VarSeq v1.8 software (GoldenHelix) for variant annotation and prioritization. The variants were filtered based on the quality and frequency criteria: coverage (>100), genotype quality score cutoff (GQS>50), variant base in at least 5% of reads, variant base present in at least 2 reads in each direction, homopolymer-length error<5, absence of genetic variants in population databases (ExAC; NHLBI-ESP; 1000 Genomes Project) or minor allele frequency (MAF)≤0.01%.

Subsequently, variants were selected based on their effect on protein expression, with the following being considered: 1) variants described in the COSMIC database; 2) loss-of-function variants - Frameshift variants-nucleotide insertions/deletions, gain/loss of stop codons, splice site alterations); or 3) missense variants (in-frame insertions/deletions, amino acid exchange) predicted as possibly pathogenic in at least three of six prediction programs used (SIFT, PolyPhen, MutationTaster, MutationAssessor, FATHMM, FATHMM-MKL) and occurring in oncogenes or tumor suppressor genes in OncoMD database. Variants not previously described in the COSMIC database were visually inspected using the integrative genomics viewer (IGV) program to exclude sequencing artifacts.

Construction of genetic interaction networks was performed using Cytoscape platform version 3.7.0, which uses data from protein and genetic interactions, pathways, co-expression, co-localization, and protein domain similarity.

## RESULTS

Initially, 40 US and UCS (14 ULMS, 12 ESS, 2 ADS, and 12 UCS) and 3 ULM samples were selected from the pathology department files; however, only 23 (7 ULMS, 7 ESS, 1 ADS, 5 UCS, and 3 ULM) remained until the end of NGS analyses. Some losses occurred while performing multiplex PCR reactions (AmpliSeq™), during which we observed a high degree of fragmented DNA and many genetic artifacts in several samples. These issues are expected since tissue processing for paraffin inclusion and long storage time causes damage to the DNA structure (integrity). The clinical and pathological features of 40 patients with US and UCS who were enrolled in this study are summarized in Table 1.

Among the 23 samples deemed suitable for the evaluation of sequencing data, homogeneity average was 73.2%, median base coverage was 1257X, and horizontal coverage was 84.3% corresponding to 100X. Based on the NGS data, we selected point mutations with possible impacts on the function of the protein encoded by the altered gene (missense, nonsense, splice-site mutations, loss of stop codons) and small insertions and deletions (indels). Total variants detected in each sample and filtered variants for the selection of somatic alterations of interest are presented in Table 2.

An average of 1700 alterations were identified per sample (ranging from 746 to 3521), with an average of 1606 single nucleotide variants (SNVs) (ranging from 678 to 3406), 40 insertions (ranging from 23 to 77), and 55 deletions (ranging from 25 to 114). To select relevant somatic variants, a first filter was applied focusing on the quality and frequencies of these alterations. A second filter, focusing on variant functions and effects, was used to select the alterations that would be most relevant in alterations of gene functions. Collectively, in 23 samples that were evaluated, 42 LOF mutations and 111 missense mutations were detected, with a total of 153 filtered mutations, among which 66 were found in the COSMIC database (Table 2).

Among the 409 genes included in the panel, mutations were detected in 94 distinct genes, with 30 genes demonstrating mutations in more than one sample and 64 genes showing mutations in a single sample. Table 3 presents the list of genes that were mutated in more than one sample of the cohort, along with the number of mutated samples and the histological types. *TP53* (11/23 - 48%), *ATM* (5/23 - 22%), and *PIK3CA* (4/23 - 17%) were the most frequently mutated genes.

**Figure 1:**
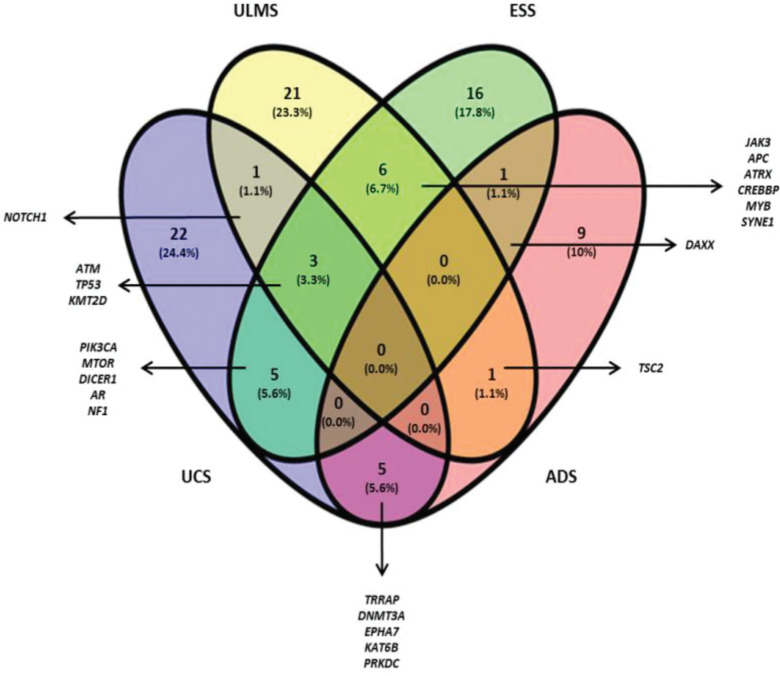
Venn diagram (Oliveros J.C, 2015) constructed using the genetic sequencing data obtained from all samples. The numbers represent shared and individual mutations for each assessed histological type.

The Venn diagram (Figure 1) shows the shared and individual (specific) mutations of each malignant histological subtype evaluated (ULMS, ESS, UCS, and ADS). Three shared genes were observed (*ATM*, *TP53*, and *KMT2D*) among the ULMS, ESS, and UCS samples. Nineteen genes were shared between 2 types of tumors, and 68 genes were mutated in a single type. Among them, 6 genes were mutated in more than one sample of the same histological subtype, namely, *PDE4DIP* (3 ESS samples), *ITGA10*, and *DST* (2 ESS samples), *NOTCH2,* and *HER2* (2 ULMS samples), and *ERBB4* (2 UCS samples). Quantitatively, this analysis shows similarities in the mutational profiles of ULMS and ESS, with 6 mutated genes in common (6.7%) between both subtypes. In the genes *JAK3*, *APC*, *ATRX*, *CREBBP*, *MYB*, and *SYNE1*, most of the mutations were characterized as missense mutations; however, in the *SYNE1* gene, the two mutations observed in ULMS and ESS samples were determined as LOF mutations (c.352C>T and c.8565G>A, respectively). In addition, mutations in the *TRRAP*, *DNMT3A*, *EPHA7*, *KAT6B*, and *PRKDC* genes indicate that UCS and ADS may exhibit molecular similarities.

Table 4 summarizes the genes with the most frequent alterations (mutations in 2 or more samples, or with 2 mutations in the same sample), the types of mutations, and their position. Alterations in the respective proteins are also indicated, along with the combined effect of these alterations (Missense or LOF) and DNA (c.), and protein (p.) nomenclatures. Their nomenclature can be used for database searches. The descriptions of the 153 potentially somatic variants are listed in Table S2. UCS5, ULMS52, ESS58107, and ADS2 samples demonstrated the highest number of mutations (UCS5 with 20 mutations in 19 genes; ULMS52 with 11 mutations in 10 genes; ESS58107 with 10 mutations in 10 genes, and ADS2 with 16 mutations in 16 genes). Samples with the lowest number of mutations were ULMS50b with 1 mutation in ALK, ESS4 with 2 mutations (*ATM* and *CREBBP*), and ULM119 (benign tissue) with 2 mutations (*MET* and *PDGFB*). 

**Figure 2:**
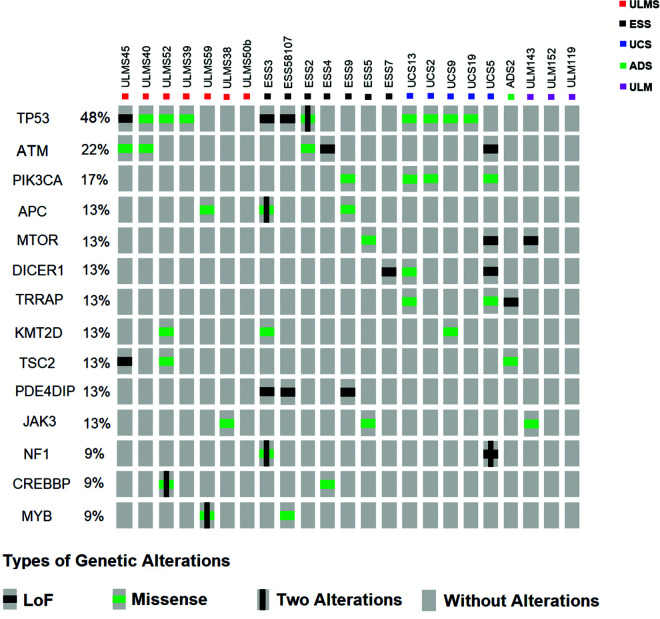
Distribution of mutations in samples and their biological effects. The figure was constructed using the OncoPrinter from cBioPortal for Cancer Genomics database (http://www.cbioportal.org/). Each gray rectangle represents a sample according to the sequence indicated at the top. Genes with the highest frequency of alterations are shown. Captions for each type of alteration (Loss of function - Black Square; Missense - Green Square; Two alterations in the same gene - vertical line [modified by authors]; No alteration - gray rectangle) are indicated.

Based on the data described in Table 4, we selected genes with more than three mutations in our cohort to submit to the OncoPrinter visualization tool (cBioPortal - http://www.cbioportal.org/). Figure 2 shows the percentage of patients demonstrating mutations in each gene, distribution, and the types of mutations observed in each sample. The highest frequency of gene mutations was observed in TP53 (48%) with the highest frequency of missense-type mutations (3 ULMS, 1 ESS, and 4 UCS samples). *ATM* mutations were observed in 22% of the samples, with 3 missense-type mutations (2 ULMS and 1 ESS) and 2 LOF-type mutations (1 ESS and 1 UCS). *PIK3CA* appeared to be the third most mutated gene (17%) present in 3 UCS samples, with most of the mutations determined as the missense-type. *APC*, *MTOR*, *DICER1*, *TRRAP*, *KMT2D*, *TSC2*, *PDE4DIP*, and *JAK3* showed a 13% mutational frequency. LOF mutations in *PDE4DIP* was found exclusively/specificaly in the ESS samples. *NF1*, *CREBBP*, and *MYB* demonstrated a 9% mutational frequency. Missense mutations in *CREBBP* and *MYB* were associated with ULMS and ESS (4 mutations in ULMS and 2 in ESS).

**Figure 3:**
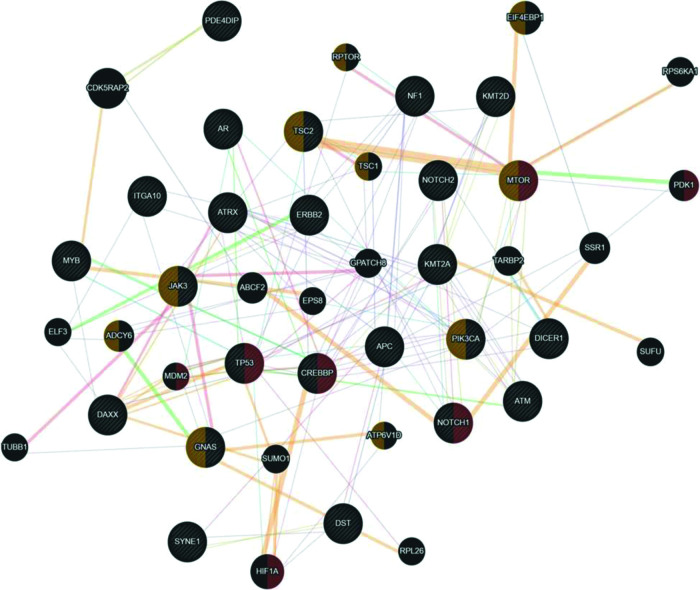
Interaction network of mutated genes in the histological types of pure sarcomas (ULMS - ESS) prepared by the Cytoscape 3.7.0 platform. The network shows patterns of predicted interaction (orange); physical interactions (red); co-expression (violet); shared proteins domains (yellow); co-localization (blue), and genetic interaction (green). Red-labeled genes have a function associated with the cellular response to hypoxia and yellow-labeled genes have a function associated with the cellular response to the peptide hormone stimulus. The genes that were inserted to perform the analysis are shown with cross-hatched circles of a uniform size. The relevant genes are shown with solid circles whose size is proportional to the number of interactions. The reported link weights are indicated visually by line thickness.

Since uterine sarcomas are histologically classified into two primary subtypes, we used the same classification to study the association of the mutated genes with pure sarcomas (ULMS - ESS) and mixed tumors (UCS - ADS). Figure 3 shows the association of the mutated genes in the group of tumors classified as pure (ULMS and ESS). According to the Cytoscape platform (20), many genes demonstrating mutations in these histological subtypes exhibit functions associated with the cellular response to hypoxia (*MTOR*, *PDK1*, *MDM2*, *TP53*, *CREBBP*, *NOTCH1,* and *HIF1A*) and peptide hormone stimulus (*EIF4EBP1*, *RPTOR*, *TSC2*, *TSC1*, *MTOR*, *JAK3*, *ADCY6*, *PIK3CA*, *GNAS,* and *ATP6V1D*). 

**Figure 4:**
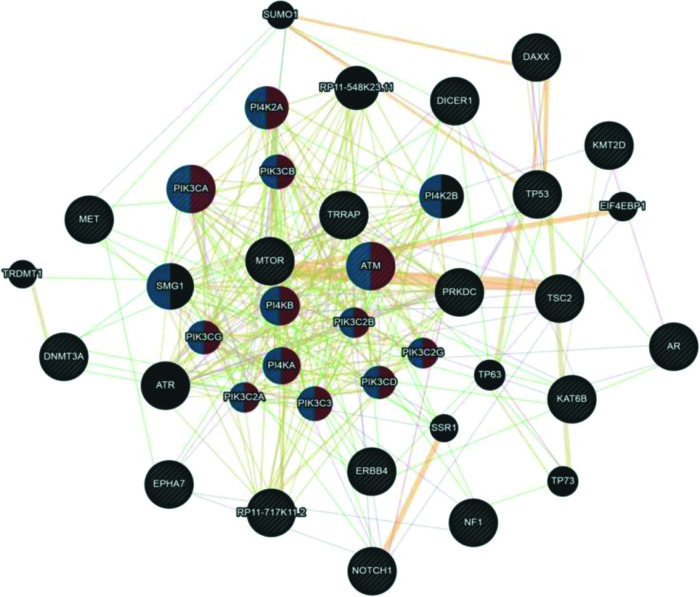
Interaction network of mutated genes in mixed tumors (UCS - ADS) prepared by the Cytoscape 3.7.0 platform. The network shows patterns of predicted interaction (orange); physical interactions (red); co-expression (violet); shared proteins domains (yellow); co-localization (blue) and genetic interaction (green). Red-labeled genes have a function associated with phosphatidylinositol kinase activity and blue-labeled genes have a function associated with the glycerophospholipid metabolic process. The genes that were inserted to perform the analysis are shown with cross-hatched circles of a uniform size. The relevant genes are shown with solid circles whose size is proportional to the number of interactions. The reported link weights are indicated visually by line thickness.

Although UCS is no longer classified as uterine sarcoma but as metaplastic carcinoma, we included this tumor group in the analysis shown in Figure 4. Here, we associated UCS - ADS owing to their mixed histologies (epithelial and mesenchymal components) and also because many retrospective studies on the US still include UCS in their available samples. According to the Cytoscape platform (20), many mutated genes in these tumors have functions associated with phosphatidylinositol kinase activity (*PI4K2A*, *PIK3CA*, *PIK3CB*, *ATM*, *PI4KB*, *PIK3CG*, *PIK3C2B*, *PI4KA*, *PIK3C2A*, *PIK3C3*, *PIK3C2G,* and *PIK3CD*) and glycerophospholipid metabolic process (*PI4K2A*, *PIK3CA*, *PIK3CB*, *ATM*, *PI4KB*, *PIK3CG*, *PIK3C2B*, *PI4KA*, *PIK3C2A*, *PIK3C3*, *PIK3C2G*, *PIK3CD, PI4K2B,* and *SMG1)*. 

Collectively, our results indicate that despite the molecular heterogeneity demonstrated by USs and UCSs, they share similarities in their mutational profiles. In addition, genetic interaction networks indicate that alterations in functions associated with hypoxia, response to peptide hormone stimulus in ULMSs and ESSs, and phosphatidylinositol kinase activity and glycerophospholipid metabolic process in UCS and ADS can influence the carcinogenic process of these tumors. Considering that NGS technology can provide a reliable molecular portrait of neoplasms quickly and cost-effectively (21), these results open new avenues for research and consequently, may positively impact the clinical management of patients with such tumors.

## DISCUSSION

In this study, we performed a mutational screening of the samples collected from patients with USs and UCSs. We employed a panel of 409 genes for the screening. Initially, we focused on the mutated genes shared among more than one histological subtype of US. We initiated our analyses with 40 samples, but owing to the quality of the FFPE material, certain losses reduced the number of samples to 23. Considering the published reports on sarcomas, the number of samples was sufficient for this type of population mutational screening. In UCS and ESS samples, we identified mutations in genes that demonstrated alterations in previous studies conducted for examining other tumors, such as *PIK3CA*, *DICER1*, *AR*, and *NF* (22). Although the role of these genes is known in different cancers, their role in the tumorigenesis of USs and USCs is not fully understood.

*The PIK3CA* gene encodes the p110α protein, the catalytic subunit of PI3K, which controls the growth, division, survival, movement, and structure of cells. Many studies have demonstrated the importance of *PIK3CA* mutation in mediating tumorigenesis via increased *PI3K*/*AKT*/*mTOR* signaling (23,24). While investigating druggable molecular targets in uterine sarcomas, Cuppens et. al (25) identified *PI3K*/*MTOR* as a potential target in 26% of cases, which were primarily ULMS, HG-ESS, and undifferentiated uterine sarcomas. Here, we included eight samples of ESS. Seven of these were characterized as HG-ESS, consistent with the molecular findings described in previous reports published for these tumors. *DICER1* is critical for the regulation of expression of several miRNAs. The *DICER1* gene is highly conserved among various species, indicating that mutations may compromise its function and might be involved in the onset of tumors (26). Previous reports published by our group (2,27) demonstrated the regulation of microRNAs associated with several oncogenic pathways, including *DICER1.* Mutations in *NF1* have already been demonstrated in soft-tissue sarcomas (myxofibrosarcomas and pleomorphic liposarcomas) (28). The expression of the androgen receptor (AR) seems to be associated with a better prognosis in patients with ESS. *AR* expression is higher in pre-malignant lesions and low-grade tumors (LG-ESS) (29). These findings may explain why AR expression is low in ULMS, which is an extremely aggressive tumor (30). However, the effects of the mutations observed in this gene need to be further investigated for US.

It is important to note that *NOTCH1* was the unique gene that shared mutations in the UCS and ULMS. Similarly, mutations in the *DAXX* gene have also been observed in the cases of ESS/ADS and ULMS/ADS, which share mutations in *TSC2*. Thus, our results suggest that besides exhibiting a similar tumor microenvironment, USs and UCSs also share genetic alterations. This observation is relevant to the understanding of the onset and evolution of these tumors. Furthermore, ULMS cases originating from ULMs have been reported; however, this hypothesis has not been proven yet (31,32). Our study showed that mutations in *KMT2A* were exclusively observed in ULMS and ULM. The c.3019G>T variant appears to be related to the Wiedemann-Steiner syndrome and Kabuki syndrome (33,34).

We attempted to identify specific genes for each type of tumor, establishing individual signatures. Despite the heterogeneity, we were able to identify six specific genes for three of the histological types evaluated in this study. In ESS samples, we observed variants in the *PDE4DIP* (c.214C>T and c.2494delC), *ITGA10* (c.2104G>A), and *DST (*c.16429C>T) genes. The variant *PDE4DIP* c.214C>T is described in the COSMIC database (35) and was first observed in papillary thyroid carcinoma. Mutations in this gene are described in several tumors, such as breast cancer as well as the cancers of the endometrium, cervix, ovaries, and urinary tract. The protein encoded by the PDE4DIP gene is responsible for binding 4D phosphodiesterase to the Golgi complex. Alterations in this gene may cause a myeloproliferative disorder associated with eosinophilia (36). Despite the information available in databases and the literature, its typical role in tumor biology remains unknown.

In UCS, we observed two variants of *ERBB4* (c.782A>C and c.2513G>A). The variant *ERBB4* c.2513G>A is described in the COSMIC database (35) as pathogenic (score 0.99) and has already been observed in hormone receptor-positive breast cancer, large bowel adenocarcinoma, malignant melanoma, and gastroesophageal junction adenocarcinoma. The role of *ERBB4* as a tumor progression factor is not fully elucidated. However, this gene is known to be overexpressed and/or mutated in several solid tumors (37). The monoclonal antibody *ERBB4* therapy is effective in breast, lung, and prostate cancer cells *in vitro* and *in vivo* (38). Specific and detailed studies may demonstrate new opportunities for the development of therapies targeting these tumors.

Mutations in *NOTCH2* and *HER2* have also been observed exclusively in ULMS. All variants are described in the COSMIC database (35). c.6094C>A mutation of *NOTCH2* is considered to be pathogenic (score 0.97) and is described in diffuse large B cell lymphoma and pancreatic ductal adenocarcinoma (PDAC). The *NOTCH2* c.7223T>A variant is also pathogenic (score 0.85) and has already been described in meningioma, a primary non-malignant CNS tumor (39). *HER2* also presented two pathogenic variants in ULMS: c.236A>C and c.2446C>T. The c.236A>C variant has already been described in meningothelial meningioma and is associated with IL-6 signaling pathways and DNA damage response. The c.2446C>T mutation has been observed in large bowel adenocarcinoma and transitional cell carcinoma of the urinary system. Persistent *NOTCH2* signaling is largely associated with poor clinical prognosis. In addition, it increases resistance to chemotherapy and radiotherapy, making these cancers less sensitive to treatment (40). *HER2* mutations have emerged as therapeutic targets for a variety of tumors. Anti- HER2 therapies are effective against breast, lung, and cervical cancers (41).

In this study, we were able to identify several mutations that contribute to a better understanding of the biology of USs and UCSs. Even with the limitations associated with rare tumors, we identified genetic alterations that might act as potential target markers for precision medicine-based approaches upon validation in larger cohorts. To date, there is no precise preoperative diagnostic test for these tumors. Although rare, such tumors are very aggressive and associated with a poor prognosis. Thus, even with small cohorts, the molecular profiling of USs and UCSs is extremely important to identify the changes driving the development of these tumors and provide powerful tools for diagnostic and prognostic tests as well as adequate treatment alternatives. Our study is the first DNA-sequencing study to investigate all histological types of USs and UCSs together and is an insightful contribution for defining the mutational repertoire of these rare tumors.

## CONCLUSIONS

Using a platform to profile mutations in a panel of 409 genes, we identified that *TP53*, *ATM*, *PIK3CA*, *APC*, *MTOR*, *DICER1*, *TRRAP*, *KMT2D*, *TSC2*, *PDE4DIP*, and *JAK3* are the most frequently mutated genes in USs and UCSs. Considering common mutations among the different tumor types being evaluated, the *TP53* (4 UCS/4 ULMS/3 ESS), *ATM* (2 ULMS/2 ESS/1 UCS), and *KMT2D* (1 UCS/1 ULMS/1 ESS) genes could be indicators of similarities in neoplastic progression. As specific signature genes, ESS exhibited mutations in the *PDE4DIP*, *IGTA10*, and *DST* genes. UCS showed mutations in the *ERBB4* gene, and ULMS demonstrated exclusive alterations in the *NOTCH2* and *HER2* genes. Mutations in the *KMT2A* gene were observed exclusively in ULM and ULMS samples, and therefore, are potentially involved in the malignant transformation process. According to the Cytoscape platform, many genes that were mutated in the ULMS and ESS samples exhibit functions associated with the cellular response to hypoxia and peptide hormone stimulus. In UCS and ADS, most altered genes exhibit functions associated with phosphatidylinositol kinase activity and glycerophospholipid metabolic process. More studies should be conducted with a larger number of samples and functional analyses. However, the current screening contributes to the characterization of the complex genetic profile of USs and USCs.

## AUTHOR CONTRIBUTIONS

Da Costa LT and Dos Anjos LG were responsible for study conceptualization, literature organization and paper elaboration. Kagohara LT collaborated in analyses of data, manuscripts and reviews. Torrezan GT and De Paula CAA contributed to the study execution. Baracat EC and Carraro DM provided intellectual support. Carvalho KC analyzed the literature, critically reviewed the manuscript, supervised the research and developed the original idea.

## Figures and Tables

**Table 1:** Clinical and pathological features of US and UCS patients (n=40).

Variables	Categories	US/UCS n (%)
Age	>50 years	33 (82)
	≤50 years	7 (18)
	N.A.	0 (0)
Postmenopausal Bleeding	Yes	22 (55)
	No	13 (33)
	N.A.	5 (12)
Adjuvant Treatment	No	8 (20)
	RT	19 (47)
	CT	8 (20)
	RT+CT	5 (13)
	N.A.	0 (0)
Metastasis or Recurrence	Yes	22 (55)
	No	14 (35)
	N.A.	4 (10)
Status	Alive	11 (27)
	Death	23 (58)
	Loss of follow-up	6 (15)
	N.A.	0 (0)

radiotherapy (RT); chemotherapy (CT); not available (NA); uterine sarcomas (US).

*ULM samples were not included owing to their benign characterization.

**Table 2:** Total variants obtained after filtering performed to increase the specificity of NGS results (higher stringency).

	General (pre-filters)				Selected Variants		
Samples	Total	SNV	Insertions	Deletions	LOFs	Missense	Cosmic
ESS 2	2347	2257	40	50	1	6	5
ESS 3	1551	1473	31	47	2	6	4
ESS 4	1249	1162	36	51	1	1	1
ESS 5	1416	1324	36	56	0	4	3
ESS 7	1494	1397	40	57	2	2	1
ESS 9	1421	1343	35	43	1	6	5
ESS 10	1440	1329	35	76	4	6	6
UCS 2	1332	1223	47	62	3	4	4
UCS 5	1362	1271	42	49	7	13	7
UCS 9	1972	1884	42	46	1	6	4
UCS 13	1234	1150	36	48	1	6	4
UCS 19	1604	1516	33	55	1	3	3
ULMS 38	746	678	43	25	0	7	4
ULMS 39	1768	1688	34	46	1	2	2
ULMS 40	1296	1193	42	61	2	3	1
ULMS 45	2004	1921	36	47	2	3	1
ULMS 52	2806	2746	23	37	0	11	6
ULMS 50	2842	2651	77	114	0	1	0
ULMS 59	2132	1968	76	88	4	6	2
ADS 2	3521	3406	41	74	6	10	0
ULM 119	1298	1201	37	60	1	1	0
ULM 143	981	919	33	29	1	2	2
ULM 152	1297	1237	25	35	1	2	1

*Endometrial stromal sarcoma (ESS); Uterine carcinosarcoma (UCS); Uterine leiomyosarcoma (ULMS); Adenocarcinoma (ADS); Uterine leiomyoma (ULM).Single nucleotide variant (SNV); Loss of function (LOFs); Catalogue of Somatic Mutations in Cancer (COSMIC).

**Table 3:** Gene mutations observed in more than one sample and histological subtypes.

Gene	Mutated samples n (%)	Histological Types (ULMS/ESS/UCS/ADS/ULM)
*TP53*	11 (48%)	4 ULMS, 3 ESS, 4 UCS
*ATM*	5 (22%)	2 ULMS, 2 ESS, 1 UCS
*PIK3CA*	4 (17%)	1 ESS, 3 UCS
*KMT2D*	3 (13%)	1 ULMS, 1 ESS, 1 UCS
*MTOR*	3 (13%)	1 ESS, 1 UCS, 1 ULM
*JAK3*	3 (13%)	1 ULMS, 1 ESS, 1 ULM
*APC*	3 (13%)	1 ULMS, 2 ESS
*DICER1*	3 (13%)	1 ESS, 2 UCS
*TRRAP*	3 (13%)	2 UCS, 1 ADS
*TSC2*	3 (13%)	2 ULMS, 1 ADS
*PDE4DIP*	3 (13%)	3 ESS
*AR*	2 (9%)	1 ESS, 1 UCS
*ATRX*	2 (9%)	1 ULMS, 1 ESS
*CREBBP*	2 (9%)	1 ULMS, 1 ESS
*DNMT3A*	2 (9%)	1 UCS, 1 ADS
*EPHA7*	2 (9%)	1 UCS, 1 ADS
*KAT6B*	2 (9%)	1 UCS, 1 ADS
*KMT2A*	2 (9%)	1 ULMS, 1 ULM
*MET*	2 (9%)	1 UCS, 1 ULM
*MYB*	2 (9%)	1 ULMS, 1 ESS
*NOTCH1*	2 (9%)	1 ULMS, 1 UCS
*PRKDC*	2 (9%)	1 UCS, 1 ADS
*SYNE1*	2 (9%)	1 ULMS, 1 ESS
*NF1*	2 (9%)	1 ESS, 1 UCS
*NOTCH2*	2 (9%)	2 ULMS
*HER2*	2 (9%)	2 ULMS
*ERBB4*	2 (9%)	2 UCS
*DAXX*	2 (9%)	1 ESS, 1 ADS
*ITGA10*	2 (9%)	2 ESS
*DST*	2 (9%)	2 ESS

**Table 4:** Most common mutations observed in the study, their chromosomal positions, effects, and nomenclature.

Sample	Chr:Pos	Gene	HGVS c.	HGVS p.	Effect
UCS2	3:178921549	*PIK3CA*	c.1031T>C	p.Val344Ala	Missense
	6:94120318	*EPHA7*	c.733G>A	p.Ala245Thr	Missense
	7:116339356	*MET*	c.218T>A	p.Leu73Ter	LOF: stop - gained
	8:48776121	*PRKDC*	c.5586delT	p.Phe1862Leufs	LOF: frameshift
	17:7577547	*TP53*	c.734G>T	p.Gly245Val	Missense
UCS5	1:11227575	*MTOR*	c.4254-1G>A	r.spl?	LOF: splice - acceptor
	3:178952085	*PIK3CA*	c.3140A>G	p.His1047Arg	Missense
	10:76735809	*KAT6B*	c.1714C>T	p.Arg572Cys	Missense
	11:108114777	*ATM*	c.594C>A	p.Cys198Ter	LOF: stop - gained
	14:95572101	*DICER1*	c.3007C>T	p.Arg1003Ter	LOF: stop - gained
	17:29588751	*NF1*	c.4600C>T	p.Arg1534Ter	LOF: stop - gained
	17:29665110	*NF1*	c.6772C>T	p.Arg2258Ter	LOF: stop - gained
	2:25469168	*DNMT3A*	c.1290T>G	p.Asn430Lys	Missense
	2:212587219	*ERBB4*	c.782A>C	p.Gln261Pro	Missense
	7:98513427	*TRRAP*	c.2281C>T	p.Arg761Trp	Missense
	X:66766207	*AR*	c.1219C>T	p.Arg407Cys	Missense
UCS9	9:139391355	*NOTCH1*	c.6836C>T	p.Ala2279Val	Missense
	12:49444719	*KMT2D*	c.2747C>T	p.Pro916Leu	Missense
	17:7578442	*TP53*	c.488A>G	p.Tyr163Cys	Missense
UCS13	3:178916854	*PIK3CA*	c.241G>A	p.Glu81Lys	Missense
	14:95574253	*DICER1*	c.2614G>A	p.Ala872Thr	Missense
	17:7577534	*TP53*	c.747G>T	p.Arg249Ser	Missense
	7:98609947	*TRRAP*	c.11549G>A	p.Arg3850His	Missense
UCS19	2:212295800	*ERBB4*	c.2513G>A	p.Arg838Gln	Missense
	17:7577580	*TP53*	c.701A>G	p.Tyr234Cys	Missense
ULMS38	1:120458122	*NOTCH2*	c.7223T>A	p.Leu2408His	Missense
	17:37864584	*HER2*	c.236A>C	p.Glu79Ala	Missense
	19:17937659	*JAK3*	c.3268G>A	p.Ala1090Thr	Missense
ULMS39	17:7577545	*TP53*	c.736A>G	p.Met246Val	Missense
ULMS40	11:108139268	*ATM*	c.2770C>T	p.Arg924Trp	Missense
	17:7577120	*TP53*	c.818G>A	p.Arg273His	Missense
	17:37881117	*HER2*	c.2446C>T	p.Arg816Cys	Missense
	X:76891445	*ATRX*	c.4660A>T	p.Arg1554Ter	LOF: stop - gained
ULMS45	11:108160506	*ATM*	c.4414T>G	p.Leu1472Val	Missense
	17:7578290	*TP53*	c.560-1G>C	r.spl?	LOF: splice - acceptor
	16:2135281	*TSC2*	c.4620C>A	p.Tyr1540Ter	LOF: stop - gained
ULMS52	1:120459251	*NOTCH2*	c.6094C>A	p.His2032Asn	Missense
	9:139400980	*NOTCH1*	c.4013C>T	p.Ala1338Val	Missense
	11:118377142	*KMT2A*	c.10535C>T	p.Pro3512Leu	Missense
	12:49416396	*KMT2D*	c.16315C>T	p.Arg5439Trp	Missense
	16:2130319	*TSC2*	c.3551C>T	p.Ala1184Val	Missense
	16:3779521	*CREBBP*	c.5527T>C	p.Cys1843Arg	Missense
	16:3790470	*CREBBP*	c.4063G>A	p.Gly1355Arg	Missense
	17:7574017	*TP53*	c.1010G>A	p.Arg337His	Missense
ULMS59	5:112173857	*APC*	c.2566C>T	p.Arg856Cys	Missense
	6:135511289	*MYB*	c.331G>A	p.Gly111Ser	Missense
ULMS59	6:135539101	*MYB*	c.2269C>T	p.Arg757Trp	Missense
	6:152832196	*SYNE1*	c.352C>T	p.Arg118Ter	LOF: stop - gained
ULMS59	20:57429026	*GNAS*	c.706G>A	p.Asp236Asn	Missense
	20:57480457	*GNAS*	c.2381A>C	p.Lys794Thr	Missense
ESS2 (LG-ESS)	6:152706896	*SYNE1*	c.8565G>A	p.Trp2855Ter	LOF: stop - gained
	11:108175463	*ATM*	c.5558A>T	p.Asp1853Val	Missense
	17:7577121	*TP53*	c.817C>T	p.Arg273Cys	Missense
ESS2	17:7577139	*TP53*	c.799C>T	p.Arg267Trp	Missense
ESS3	1:145015874	*PDE4DIP*	c.214C>T	p.Arg72Ter	LOF: stop - gained
	5:112154777	*APC*	c.1048T>C	p.Ser350Pro	Missense
	5:112162855	*APC*	c.1459G>A	p.Gly487Arg	Missense
	6:56328464	*DST*	c.16429C>T	p.Arg5477Trp	Missense
	12:49418436	*KMT2D*	c.15977T>C	p.Leu5326Pro	Missense
	17:7578176	*TP53*	c.672+1G>A	r.spl?	LOF: splice - donor
	17:29556250	*NF1*	c.2617C>T	p.Arg873Cys	Missense
	17:29677234	*NF1*	c.7355G>T	p.Arg2452Leu	Missense
ESS4	11:108141990	*ATM*	c.2934delT	p.Leu979Cysfs	LOF: frameshift
	16:3820773	*CREBBP*	c.2678C>T	p.Ser893Leu	Missense
ESS5	1:11217330	*MTOR*	c.4348T>G	p.Tyr1450Asp	Missense
	19:17937659	*JAK3*	c.3268G>A	p.Ala1090Thr	Missense
ESS7	6:33287248	*DAXX*	c.1885G>A	p.Val629Ile	Missense
	14:95590677	*DICER1*	c.1232C>A	p.Ser411Ter	LOF: stop - gained
ESS7	X:76939115	*ATRX*	c.1633C>G	p.Gln545Glu	Missense
ESS9	1:144906139	*PDE4DIP*	c.2494delC	p.Gln832Argfs	LOF - frameshift
	1:145536012	*ITGA10*	c.2104G>A	p.Ala702Thr	Missense
	3:178936091	*PIK3CA*	c.1633G>A	p.Glu545Lys	Missense
	5:112175711	*APC*	c.4420G>A	p.Ala1474Thr	Missense
ESS58107	1:145015874	*PDE4DIP*	c.214C>T	p.Arg72Ter	LOF: stop - gained
	1:145536012	*ITGA10*	c.2104G>A	p.Ala702Thr	Missense
	6:56328464	*DST*	c.16429C>T	p.Arg5477Trp	Missense
	6:135516944	*MYB*	c.1007C>T	p.Thr336Ile	Missense
	17:7578176	*TP53*	c.672+1G>A	r.spl?	LOF: splice - donor
	X:66863156	*AR*	c.1675A>T	p.Thr559Ser	Missense
ADS2	2:25467477	*DNMT3A*	c.1599C>A	p.Tyr533Ter	LOF: stop - gained
	6:33288629	*DAXX*	c.959A>G	p.Gln320Arg	Missense
	6:93979315	*EPHA7*	c.1513C>A	p.Leu505Met	Missense
	7:98501128	*TRRAP*	c.1024G>T	p.Glu342Ter	LOF: stop - gained
	8:48711786	*PRKDC*	c.10279G>T	p.Glu3427Ter	LOF: stop - gained
	10:76781925	*KAT6B*	c.3308_3310delAAG	p.Glu1104del	LOF: inframe/del
	16:2138078	*TSC2*	c.5098G>T	p.Ala1700Ser	Missense
ULM119	7:116403114	*MET*	c.2429A>C	p.His810Pro	Missense
ULM143	1:11307996	*MTOR*	c.995_996dupGG	p.Leu333Glyfs	LOF: frameshift
	19:17945696	*JAK3*	c.2164G>A	p.Val722Ile	Missense
ULM152	11:118344893	*KMT2A*	c.3019G>T	p.Gly1007Cys	Missense

## Appendix

**Table S1:** Ion AmpliSeq Comprehensive Cancer Panel target gene list.

1	*ABL1*	*CBL*	*EP300*	*GATA2*	*LAMP1*	*MYD88*	*PKHD1*	*SMARCA4*	*WHSC1*
2	*ABL2*	*CCND1*	*EP400*	*GATA3*	*LCK*	*MYH11*	*PLAG1*	*SMARCB1*	*WRN*
3	*ACVR2A*	*CCND2*	*EPHA3*	*GDNF*	*LIFR*	*MYH9*	*PLCG1*	*SMO*	*WT1*
4	*ADAMTS20*	*CCNE1*	*EPHA7*	*GNA11*	*LPHN3*	*NBN*	*PLEKHG5*	*SMUG1*	*XPA*
5	*AFF1*	*CD79A*	*EPHB1*	*GNAQ*	*POT1*	*NCOA1*	*PML*	*SOCS1*	*XPC*
6	*AFF3*	*CD79B*	*EPHB4*	*GNAS*	*LPP*	*NCOA2*	*PMS1*	*SOX11*	*XPO1*
7	*AKAP9*	*CDC73*	*EPHB6*	*GPR124*	*LRP1B*	*NCOA4*	*PMS2*	*SOX2*	*XRCC2*
8	*AKT1*	*CDH1*	*ERBB2*	*GRM8*	*LTF*	*NF1*	*POU5F1*	*SRC*	*ZNF384*
9	*AKT2*	*CDH11*	*ERBB3*	*GUCY1A2*	*LTK*	*NF2*	*PPARG*	*SSX1*	*ZNF521*
10	*AKT3*	*CDH2*	*ERBB4*	*HCAR1*	*MAF*	*NFE2L2*	*PPP2R1A*	*STK11*	
11	*ALK*	*CDH20*	*ERCC1*	*HIF1A*	*MAFB*	*NFKB1*	*PRDM1*	*STK36*	
12	*APC*	*CDH5*	*ERCC2*	*HLF*	*MAGEA1*	*NFKB2*	*PRKAR1A*	*SUFU*	
13	*AR*	*CDK12*	*ERCC3*	*HNF1A*	*MAGI1*	*NIN*	*PRKDC*	*SYK*	
14	*ARID1A*	*CDK4*	*ERCC4*	*HOOK3*	*MALT1*	*NKX2-1*	*PSIP1*	*SYNE1*	
15	*ARID2*	*CDK6*	*ERCC5*	*HRAS*	*MAML2*	*NLRP1*	*PTCH1*	*TAF1*	
16	*ARNT*	*CDK8*	*ERG*	*HSP90AA1*	*MAP2K1*	*NOTCH1*	*PTEN*	*TAF1L*	
17	*ASXL1*	*CDKN2A*	*ESR1*	*HSP90AB1*	*MAP2K2*	*NOTCH2*	*PTGS2*	*TAL1*	
18	*ATF1*	*CDKN2B*	*ETS1*	*ICK*	*MAP2K4*	*NOTCH4*	*PTPN11*	*TBX22*	
19	*ATM*	*CDKN2C*	*ETV1*	*IDH1*	*MAP3K7*	*NPM1*	*PTPRD*	*TCF12*	
20	*ATR*	*CEBPA*	*ETV4*	*IDH2*	*MAPK1*	*NRAS*	*PTPRT*	*TCF3*	
21	*ATRX*	*CHEK1*	*EXT1*	*IGF1R*	*MAPK8*	*NSD1*	*RAD50*	*TCF7L1*	
22	*AURKA*	*CHEK2*	*EXT2*	*IGF2*	*MARK1*	*NTRK1*	*RAF1*	*TCF7L2*	
23	*AURKB*	*CIC*	*EZH2*	*IGF2R*	*MARK4*	*NTRK3*	*RALGDS*	*TCL1A*	
24	*AURKC*	*CKS1B*	*FAM123B*	*IKBKB*	*MBD1*	*NUMA1*	*RARA*	*TET1*	
25	*AXL*	*CMPK1*	*FANCA*	*IKBKE*	*MCL1*	*NUP214*	*RB1*	*TET2*	
26	*BAI3*	*COL1A1*	*FANCC*	*IKZF1*	*MDM2*	*NUP98*	*RECQL4*	*TFE3*	
27	*BAP1*	*CRBN*	*FANCD2*	*IL2*	*MDM4*	*PAK3*	*REL*	*TGFBR2*	
28	*BCL10*	*CREB1*	*FANCF*	*IL21R*	*MEN1*	*PALB2*	*RET*	*TGM7*	
29	*BCL11A*	*CREBBP*	*FANCG*	*IL6ST*	*MET*	*PARP1*	*RHOH*	*THBS1*	
30	*BCL11B*	*CRKL*	*FAS*	*IL7R*	*MITF*	*PAX3*	*RNASEL*	*TIMP3*	
31	*BCL2*	*CRTC1*	*FBXW7*	*ING4*	*MLH1*	*PAX5*	*RNF2*	*TLR4*	
32	*BCL2L1*	*CSF1R*	*FGFR1*	*IRF4*	*MLL*	*PAX7*	*RNF213*	*TLX1*	
33	*BCL2L2*	*CSMD3*	*FGFR2*	*IRS2*	*MLL2*	*PAX8*	*ROS1*	*TNFAIP3*	
34	*BCL3*	*CTNNA1*	*FGFR3*	*ITGA10*	*MLL3*	*PBRM1*	*RPS6KA2*	*TNFRSF14*	
35	*BCL6*	*CTNNB1*	*FGFR4*	*ITGA9*	*MLLT10*	*PBX1*	*RRM1*	*TNK2*	
36	*BCL9*	*CYLD*	*FH*	*ITGB2*	*MMP2*	*PDE4DIP*	*RUNX1*	*TOP1*	
37	*BCR*	*CYP2C19*	*FLCN*	*ITGB3*	*MN1*	*PDGFB*	*RUNX1T1*	*TP53*	
38	*BIRC2*	*CYP2D6*	*FLI1*	*JAK1*	*MPL*	*PDGFRA*	*SAMD9*	*TPR*	
39	*BIRC3*	*DAXX*	*FLT1*	*JAK2*	*MRE11A*	*PDGFRB*	*SBDS*	*TRIM24*	
40	*BIRC5*	*DCC*	*FLT3*	*JAK3*	*MSH2*	*PER1*	*SDHA*	*TRIM33*	
41	*BLM*	*DDB2*	*FLT4*	*JUN*	*MSH6*	*PGAP3*	*SDHB*	*TRIP11*	
42	*BLNK*	*DDIT3*	*FN1*	*KAT6A*	*MTOR*	*PHOX2B*	*SDHC*	*TRRAP*	
43	*BMPR1A*	*DDR2*	*FOXL2*	*KAT6B*	*MTR*	*PIK3C2B*	*SDHD*	*TSC1*	
44	*BRAF*	*DEK*	*FOXO1*	*KDM5C*	*MTRR*	*PIK3CA*	*SEPT9*	*TSC2*	
45	*BRD3*	*DICER1*	*FOXO3*	*KDM6A*	*MUC1*	*PIK3CB*	*SETD2*	*TSHR*	
46	*BRIP1*	*DNMT3A*	*FOXP1*	*KDR*	*MUTYH*	*PIK3CD*	*SF3B1*	*UBR5*	
47	*BTK*	*DPYD*	*FOXP4*	*KEAP1*	*MYB*	*PIK3CG*	*SGK1*	*UGT1A1*	
48	*BUB1B*	*DST*	*FZR1*	*KIT*	*MYC*	*PIK3R1*	*SH2D1A*	*USP9X*	
49	*CARD11*	*EGFR*	*G6PD*	*KLF6*	*MYCL1*	*PIK3R2*	*SMAD2*	*VHL*	
50	*CASC5*	*EML4*	*GATA1*	*KRAS*	*MYCN*	*PIM1*	*SMAD4*	*WAS*	

**Table S2:** Description of 153 potential somatic variants selected in 23 samples of uterine tumors.

Sample	Chr:Pos	Gene	HGVS c.	HGVS p.	Effect
UCS2	3:178921549	*PIK3CA*	c.1031T>C	p.Val344Ala	Missense
	6:94120318	*EPHA7*	c.733G>A	p.Ala245Thr	Missense
	7:116339356	*MET*	c.218T>A	p.Leu73Ter	LOF: stop - gained
	8:48776121	*PRKDC*	c.5586delT	p.Phe1862Leufs	LOF: frameshift
	15:99500303	*IGF1R*	c.3736C>T	p.Arg1246Cys	Missense
	17:7577547	*TP53*	c.734G>T	p.Gly245Val	Missense
	22:33253291	*TIMP3*	c.260delC	p.Glu88Argfs	LOF: frameshift
UCS5	1:11227575	*MTOR*	c.4254-1G>A	r.spl?	LOF: splice - acceptor
	1:27105553	*ARID1A*	c.5164C>T	p.Arg1722Ter	LOF: stop - gained
	1:65310574	*JAK1*	c.2116-2A>G	r.spl?	LOF: splice - acceptor
	3:178952085	*PIK3CA*	c.3140A>G	p.His1047Arg	Missense
	10:76735809	*KAT6B*	c.1714C>T	p.Arg572Cys	Missense
	10:97969609	*BLNK*	c.731C>T	p.Pro244Leu	Missense
	11:108114777	*ATM*	c.594C>A	p.Cys198Ter	LOF: stop - gained
	14:95572101	*DICER1*	c.3007C>T	p.Arg1003Ter	LOF: stop - gained
	17:29588751	*NF1*	c.4600C>T	p.Arg1534Ter	LOF: stop - gained
	17:29665110	*NF1*	c.6772C>T	p.Arg2258Ter	LOF: stop - gained
	19:45260400	*BCL3*	c.646C>T	p.Arg216Cys	Missense
	1:47685756	*TAL1*	c.632G>A	p.Arg211His	Missense
	2:25469168	*DNMT3A*	c.1290T>G	p.Asn430Lys	Missense
	2:212587219	*ERBB4*	c.782A>C	p.Gln261Pro	Missense
	7:98513427	*TRRAP*	c.2281C>T	p.Arg761Trp	Missense
	9:37015073	*PAX5*	c.331G>A	p.Ala111Thr	Missense
	19:11098401	*SMARCA4*	c.919C>T	p.Pro307Ser	Missense
	20:36030940	*SRC*	c.1219G>A	p.Asp407Asn	Missense
	X:44942716	*KDM6A*	c.3452A>G	p.Gln1151Arg	Missense
	X:66766207	*AR*	c.1219C>T	p.Arg407Cys	Missense
UCS9	9:139391355	*NOTCH1*	c.6836C>T	p.Ala2279Val	Missense
	10:123298226	*FGFR2*	c.628C>T	p.Arg210Ter	LOF: stop - gained
	12:49444719	*KMT2D*	c.2747C>T	p.Pro916Leu	Missense
	15:40916649	*KNL1*	c.4265G>A	p.Arg1422Gln	Missense
	17:7578442	*TP53*	c.488A>G	p.Tyr163Cys	Missense
	3:52440867	*BAP1*	c.637C>T	p.Arg213Cys	Missense
	21:39755729	*ERG*	c.1057G>A	p.Glu353Lys	Missense
UCS13	3:178916854	*PIK3CA*	c.241G>A	p.Glu81Lys	Missense
	11:71726283	*NUMA1*	c.2266G>T	p.Glu756Ter	LOF: stop - gained
	13:29001422	*FLT1*	c.1310C>T	p.Ser437Leu	Missense
	14:95574253	*DICER1*	c.2614G>A	p.Ala872Thr	Missense
	17:7577534	*TP53*	c.747G>T	p.Arg249Ser	Missense
	5:176636902	*NSD1*	c.1502A>G	p.Lys501Arg	Missense
	7:98609947	*TRRAP*	c.11549G>A	p.Arg3850His	Missense
UCS19	2:212295800	*ERBB4*	c.2513G>A	p.Arg838Gln	Missense
	9:5126715	*JAK2*	c.3323A>G	p.Asn1108Ser	Missense
	17:7577580	*TP53*	c.701A>G	p.Tyr234Cys	Missense
	17:37829120	*PGAP3*	c.900-1G>A	r.spl?	LOF: splice - acceptor
ULMS38	1:120458122	*NOTCH2*	c.7223T>A	p.Leu2408His	Missense
	6:51914991	*PKHD1*	c.2243C>T	p.Ala748Val	Missense
	16:23646942	*PALB2*	c.925A>G	p.Ile309Val	Missense
	17:5462805	*NLRP1*	c.1211G>A	p.Arg404Gln	Missense
	17:37864584	*ERBB2*	c.236A>C	p.Glu79Ala	Missense
	3:65425588	*MAGI1*	c.1234_1236delCAG	p.Gln421del	Inframe - deletion
	19:17937659	*JAK3*	c.3268G>A	p.Ala1090Thr	Missense
ULMS39	3:188327501	*LPP*	c.982C>T	p.Arg328Trp	Missense
	7:142562071	*EPHB6*	c.513_515delCTC	p.Ser176del	LOF: disruptive - inframe - del
	17:7577545	*TP53*	c.736A>G	p.Met246Val	Missense
ULMS40	2:100218031	*AFF3*	c.1310_1312delGCA	p.Ser444del	LOF: disruptive - inframe - del
ULMS40	11:108139268	*ATM*	c.2770C>T	p.Arg924Trp	Missense
	17:7577120	*TP53*	c.818G>A	p.Arg273His	Missense
	17:37881117	*ERBB2*	c.2446C>T	p.Arg816Cys	Missense
	X:76891445	*ATRX*	c.4660A>T	p.Arg1554Ter	LOF: stop - gained
ULMS45	3:128204775	*GATA2*	c.666G>C	p.Lys222Asn	Missense
	11:108160506	*ATM*	c.4414T>G	p.Leu1472Val	Missense
	12:121437187	*HNF1A*	c.1618A>G	p.Lys540Glu	Missense
	17:7578290	*TP53*	c.560-1G>C	r.spl?	LOF: splice - acceptor
	16:2135281	*TSC2*	c.4620C>A	p.Tyr1540Ter	LOF: stop - gained
ULMS50b	2:29432740	*ALK*	c.3748A>G	p.Ile1250Val	Missense
ULMS52	1:6528318	*PLEKHG5*	c.2815C>T	p.Arg939Cys	Missense
	1:120459251	*NOTCH2*	c.6094C>A	p.His2032Asn	Missense
	9:139400980	*NOTCH1*	c.4013C>T	p.Ala1338Val	Missense
	11:118377142	*KMT2A*	c.10535C>T	p.Pro3512Leu	Missense
	12:49416396	*KMT2D*	c.16315C>T	p.Arg5439Trp	Missense
	13:26978093	*CDK8*	c.1270C>T	p.Arg424Cys	Missense
	16:2130319	*TSC2*	c.3551C>T	p.Ala1184Val	Missense
	16:3779521	*CREBBP*	c.5527T>C	p.Cys1843Arg	Missense
	16:3790470	*CREBBP*	c.4063G>A	p.Gly1355Arg	Missense
	17:7574017	*TP53*	c.1010G>A	p.Arg337His	Missense
	22:36678790	*MYH9*	c.5807G>A	p.Arg1936Gln	Missense
ULMS59	5:112173857	*APC*	c.2566C>T	p.Arg856Cys	Missense
	6:135511289	*MYB*	c.331G>A	p.Gly111Ser	Missense
	6:135539101	*MYB*	c.2269C>T	p.Arg757Trp	Missense
	6:152832196	*SYNE1*	c.352C>T	p.Arg118Ter	LOF: stop - gained
	7:2946463	*CARD11*	c.3274C>T	p.Arg1092Ter	LOF: stop - gained
	18:22806393	*ZNF521*	c.1489C>T	p.Arg497Ter	LOF: stop - gained
	18:47803035	*MBD1*	c.472C>T	p.Arg158Ter	LOF: stop - gained
	20:57429026	*GNAS*	c.706G>A	p.Asp236Asn	Missense
	20:57480457	*GNAS*	c.2381A>C	p.Lys794Thr	Missense
	22:30069262	*NF2*	c.1127G>A	p.Arg376Gln	Missense
ESS2 (LG-ESS)	6:152706896	*SYNE1*	c.8565G>A	p.Trp2855Ter	LOF: stop - gained
	11:108175463	*ATM*	c.5558A>T	p.Asp1853Val	Missense
	14:81610269	*TSHR*	c.1867G>T	p.Ala623Ser	Missense
	17:7577121	*TP53*	c.817C>T	p.Arg273Cys	Missense
	17:7577139	*TP53*	c.799C>T	p.Arg267Trp	Missense
	19:3119273	*GNA11*	c.805G>A	p.Val269Ile	Missense
	22:41553308	*EP300*	c.3397C>T	p.Arg1133Trp	Missense
ESS3	1:145015874	*PDE4DIP*	c.214C>T	p.Arg72Ter	LOF: stop - gained
	5:112154777	*APC*	c.1048T>C	p.Ser350Pro	Missense
	5:112162855	*APC*	c.1459G>A	p.Gly487Arg	Missense
	6:56328464	*DST*	c.16429C>T	p.Arg5477Trp	Missense
	12:49418436	*KMT2D*	c.15977T>C	p.Leu5326Pro	Missense
	17:7578176	*TP53*	c.672+1G>A	r.spl?	LOF: splice - donor
	17:29556250	*NF1*	c.2617C>T	p.Arg873Cys	Missense
	17:29677234	*NF1*	c.7355G>T	p.Arg2452Leu	Missense
ESS4	11:108141990	*ATM*	c.2934delT	p.Leu979Cysfs	LOF: frameshift
	16:3820773	*CREBBP*	c.2678C>T	p.Ser893Leu	Missense
ESS5	1:11217330	*MTOR*	c.4348T>G	p.Tyr1450Asp	Missense
	14:51227050	*NIN*	c.1924G>A	p.Glu642Lys	Missense
	19:17937659	*JAK3*	c.3268G>A	p.Ala1090Thr	Missense
	20:41101170	*PTPRT*	c.1186G>A	p.Val396Ile	Missense
ESS7	6:33287248	*DAXX*	c.1885G>A	p.Val629Ile	Missense
	6:117710646	*ROS1*	c.1626delT	p.Phe542Leufs	LOF: frameshift
	14:95590677	*DICER1*	c.1232C>A	p.Ser411Ter	LOF: stop - gained
ESS7	X:76939115	*ATRX*	c.1633C>G	p.Gln545Glu	Missense
ESS9	1:144906139	*PDE4DIP*	c.2494delC	p.Gln832Argfs	LOF: frameshift
	1:145536012	*ITGA10*	c.2104G>A	p.Ala702Thr	Missense
	3:178936091	*PIK3CA*	c.1633G>A	p.Glu545Lys	Missense
	4:55564641	*KIT*	c.529C>T	p.Arg177Cys	Missense
	4:55976709	*KDR*	c.1116G>C	p.Glu372Asp	Missense
	5:112175711	*APC*	c.4420G>A	p.Ala1474Thr	Missense
	5:180048651	*FLT4*	c.1911C>G	p.Ser637Arg	Missense
ESS58107	1:145015874	*PDE4DIP*	c.214C>T	p.Arg72Ter	LOF: stop - gained
	1:145536012	*ITGA10*	c.2104G>A	p.Ala702Thr	Missense
	2:142567932	*LRP1B*	c.121G>A	p.Asp41Asn	Missense
	4:153332477	*FBXW7*	c.479C>T	p.Pro160Leu	Missense
	6:56328464	*DST*	c.16429C>T	p.Arg5477Trp	Missense
	6:135516944	*MYB*	c.1007C>T	p.Thr336Ile	Missense
	7:91570414	*AKAP9*	c.1A>G	p.Met1?	LOF: initiator - codon
	17:7578176	*TP53*	c.672+1G>A	r.spl?	LOF: splice - donor
	X:41056743	*USP9X*	c.4360delG	p.Gly1454Glufs	LOF: frameshift
	X:66863156	*AR*	c.1675A>T	p.Thr559Ser	Missense
ADS2	1:162748436	*DDR2*	c.2350T>C	p.Cys784Arg	Missense
	2:25467477	*DNMT3A*	c.1599C>A	p.Tyr533Ter	LOF: stop - gained
	2:209110123	*IDH1*	c.440C>A	p.Pro147His	Missense
	3:38182306	*MYD88*	c.766T>C	p.Phe256Leu	Missense
	5:131927073	*RAD50*	c.1610delA	p.Met538Trpfs	LOF: frameshift
	6:33288629	*DAXX*	c.959A>G	p.Gln320Arg	Missense
	6:93979315	*EPHA7*	c.1513C>A	p.Leu505Met	Missense
	7:98501128	*TRRAP*	c.1024G>T	p.Glu342Ter	LOF: stop - gained
	8:48711786	*PRKDC*	c.10279G>T	p.Glu3427Ter	LOF: stop - gained
	9:98209391	*PTCH1*	c.4147C>A	p.Pro1383Thr	Missense
	10:76781925	*KAT6B*	c.3308_3310delAAG	p.Glu1104del	LOF: disruptive - inframe - del
	11:106558436	*GUCY1A2*	c.2131G>T	p.Glu711Ter	LOF: stop - gained
	15:90630454	*IDH2*	c.857A>G	p.Glu286Gly	Missense
	16:2138078	*TSC2*	c.5098G>T	p.Ala1700Ser	Missense
	22:29121048	*CHEK2*	c.638T>C	p.Val213Ala	Missense
	X:53223847	*KDM5C*	c.3512A>G	p.Lys1171Arg	Missense
ULM119	7:116403114	*MET*	c.2429A>C	p.His810Pro	Missense
	22:39621795	*PDGFB*	c.659dupA	p.Lys222Glnfs	LOF: frameshift
ULM143	1:11307996	*MTOR*	c.995_996dupGG	p.Leu333Glyfs	LOF: frameshift
	9:32634260	*TAF1L*	c.1318A>G	p.Ile440Val	Missense
	19:17945696	*JAK3*	c.2164G>A	p.Val722Ile	Missense
ULM152	8:41791030	*KAT6A*	c.4708G>A	p.Asp1570Asn	Missense
	11:118344893	*KMT2A*	c.3019G>T	p.Gly1007Cys	Missense
	19:1207176	*STK11*	c.263_264insC	p.Asn90Glnfs	LOF: frameshift
